# A Simplified Liquid Chromatography-Mass Spectrometry Assay for Artesunate and Dihydroartemisinin, Its Metabolite, in Human Plasma

**DOI:** 10.3390/molecules15128747

**Published:** 2010-12-01

**Authors:** Paktiya Teja-Isavadharm, Duangsuda Siriyanonda, Raveewan Siripokasupkul, Roongnapa Apinan, Nitima Chanarat, Apassorn Lim, Srisombat Wannaying, David Saunders, Mark M. Fukuda, Robert S. Miller, Peter J. Weina, Victor Meléndez

**Affiliations:** 1 Department of Immunology and Medicine, United States Army Medical Component, Armed Forces Research Institute of Medical Sciences, 315/6 Rajvithi Road, Bangkok 10400, Thailand; 2 Department of Pharmacology, Division of Experimental Therapeutics, Walter Reed Army Institute of Research, Silver Spring, MD 20910, USA; Email: Robert.s.miller@amedd.army.mil (R.S.M.); peter.weina@us.army.mil (P.J.W.)

**Keywords:** artesunate, dihydroartemisinin, human plasma, method validation, LC-MS

## Abstract

Artesunate (AS) is a potent antimalarial that is used worldwide for the treatment of malaria. A simple method with a total run time of 12 min was developed and validated for the quantification of AS and dihydroartemisinin (DHA), its active metabolite, in human (heparinized) plasma based on one-step protein precipitation in acetonitrile using artemisinin (ARN) as an internal standard, followed by liquid chromatography with a single quadrupole mass spectrometry system connected to a C_18_ column. Peak area ratio responses were fitted to the 2^nd^-order curve type, polynomial equation with weighting (1/concentration) over a quantification range between 3.20/5.33–3,000/5,000 nM (1.23/1.52–1153/1422 ng/mL) of AS/DHA showing linearity with very good correlation (r^2^ > 0.999). Single ion recordings of 5 µL injections of plasma extracts allowed for limits of detection of 1.02 nM (0.39 ng/mL) for AS and 0.44 nM (0.13 ng/mL) for DHA. The inter-assay and intra-assay accuracy and precision of the method was very good with an inaccuracy of ±12.4% and coefficients of variation of ≤10.7% at all tested concentrations. The recovery of the analytes from plasma was ≥95%. Other commonly used antimalarials including mefloquine, quinine, and chloroquine, did not interfere with the analysis. Post-preparative tests over 24 h in an autosampler (10 °C) showed that the DHA response was only 2.1% of AS from auto-hydrolysis, and β-DHA was the major, stable epimer that was used for quantification of DHA. In contrast, α-DHA increased steadily up to 600%. Artesunate and DHA in plasma were stable through three freeze/thaw cycles for up to 6 h at room temperature and up to one year at -80 °C.

## 1. Introduction

*Plasmodium falciparum* causes almost all of the >1.5 million world-wide deaths from malaria each year [1,2]. Parenteral artesunate is the treatment of choice for severe malaria in many countries as recommended by the WHO [2,3] and is effective against chloroquine resistant strains of *P. falciparum* malaria. Artesunate {butanedioic acid, [3*R*-(3α,5αβ,6β,8aβ, 9α,10α,12β,12a R*)] -mono(decahydro-3,6,9-trimethyl-3,12-epoxy-12H-pyrano[4,3-j]-1,2-benzodioxepin-10-yl) ester} is a semi-synthetic derivative of artemisinin {(3*R*,5a*S*,6*R*,8a*S*,9*R*,12*S*,12a*R*)-octahydro-3,6,9-trimethyl-3,12-epoxy-12*H* pyrano[4,3-*j*]-1,2-benzodioxepin-10(3*H*)-one} which was isolated in 1972 from the plant qinghao (*Artemisia annua*). Artemisinin derivatives are nitrogen-free sesquiterpenes with endo-peroxide linkages that are responsible for the antimalarial activity. Artemisinins are thought to cause free radical damage to parasite membranes through binding with malarial proteins by scavenging iron and heme, both essential for parasite survival [4]. Recently, artemisinin (ARN) was reported to inhibit *P. falciparum* PfATP6, a calcium dependent ATPase [5]. Artesunate (AS) is a safe drug with few side effects and has a global marketplace [6]. Due to high relapse rates of short-course artesunate monotherapy, patients with uncomplicated malaria are generally treated with AS partnered with a long-acting antimalarial such as mefloquine or piperaquine [7]. 

**Figure 1:**
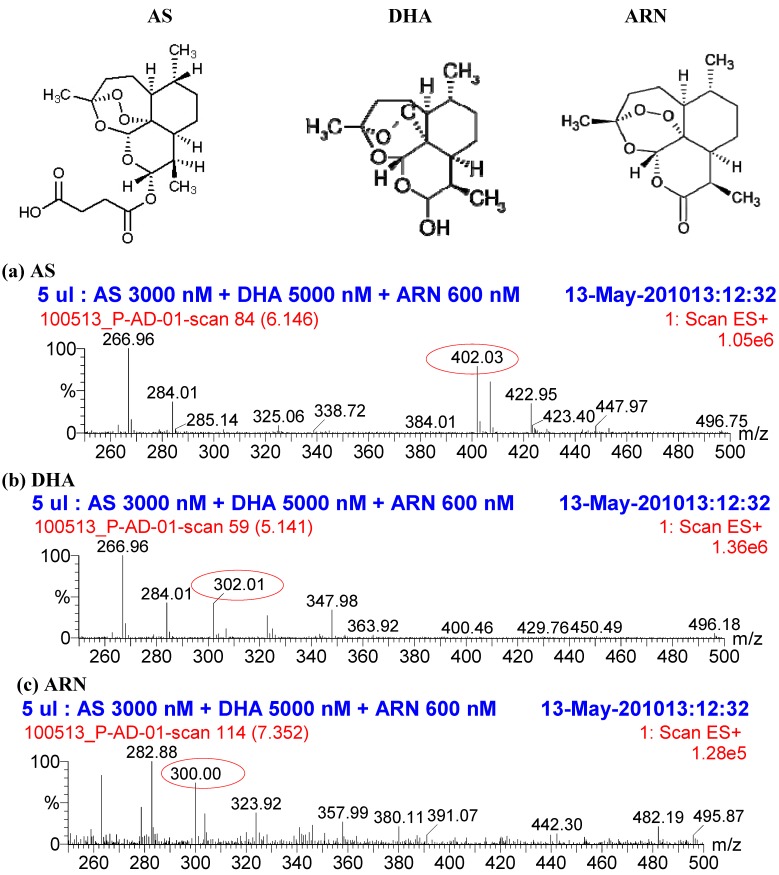
Full scan mass spectrum of (**a**) AS, (**b**) DHA and (**c**) ARN internal standard extracted from human plasma. Artesunate and DHA were detected by LC-MS using electrospray ionization in positive ion mode and single ion recording. Both analytes and IS formed ammonium adducts [M+NH_4_]^+^ having unique m/z ratios of 402, 302 and 300, respectively; m/z ratios at 267 and 284 are common fragments for both AS and DHA.

As a hemisuccinate ester of dihydroartemisinin (DHA), AS is rapidly biotransformed to DHA {[3R-(3α,5aβ,6β,8aβ,9α,10α,12β,12aR*)]-decahydro-10-hydroxy-3,6,9-trimethyl-3,12-epoxy-12H-pyrano[4.3-j]-1,2-benzodioxepin} which is an active metabolite (Figure 1). The rate of transformation and the bioavailability depend on the route of administration, and multiple effective routes including intravenous, intramuscular, oral, and rectal give the drug broad clinical applications. Previously, the standard bioanalytical method to measure AS and DHA from clinical samples relied on HPLC with reductive electrochemical detection systems; however, these were complicated and required large sample volumes [8,9]. Currently, most research groups use liquid chromatography with mass spectrometry detection (LC-MS or LC-MS/MS), a highly sensitive method with much smaller sample requirements. However, the extraction methods for AS and DHA were either liquid-liquid or solid phase extraction (SPE) which are complex and time-consuming [8,9,10,11,12] or otherwise expensive, as in the case of 96-well plate-based SPE [13]. Although rapid, this method requires a 96-well autosampler, not commonly available in most pharmacology laboratories. 

Here we report the development of a simple and rapid method for the quantification of both AS and DHA simultaneously from human plasma with ARN as internal standard (IS), based on one-step protein precipitation in acetonitrile (ACN) followed by LC-MS analysis. This method proved to be sensitive and selective with a wide detection range and a low detection limit. We present data on long-term stability of AS and DHA stored in plasma samples, stability of DHA α/β epimers as well as potential interactions with other antimalarials commonly used in the treatment of malaria such as chloroquine and quinine and AS partner drugs with long elimination half-life such as mefloquine.

## 2. Results and Discussion

### 2.1. Quantification of AS and its metabolite DHA

We originally developed a novel assay for the quantification of AS and its metabolite DHA in heparinized plasma samples using an external standard method. An internal standard was not used because the simple one step protein precipitation with two volumes 100% ACN consistently yielded over 97% recovery of the analytes across the analytical range, there was no drug loss and no need to concentrate samples in the low end of the analytical range as in the case of liquid-liquid or solid-phase extractions. LC-MS system reproducibility was ascertained by system suitability and accuracy of QC samples determinations. Volumes for standard curves were accurately pipetted using the same calibrated pipettes with the same accuracy and precision used for pipetting analytical samples. A full validation of the method was performed with acceptable results based on FDA and ICH guidance for bioanalytical method validation [14,15]. The method was applied to measuring clinical samples from African patients following intravenous (IV) administration of AS for the treatment of severe malaria. 

When patients’ samples are used, unexpected components (*i.e.* haemolysis product from poor blood collection or from parasites) may alter the stability of the analytes. Therefore, using an appropriate internal standard (*i.e.* stable isotopic labeled analytes, or a compound with a structure similar to the analytes) ensures that any alteration of the analytes will have the same effect on the IS. As a result, the use of constant amount of IS added to all samples can correct for those unexpected effects on the quantification of the analytes. Therefore, we performed a partial validation using ARN as IS at 600 nM and subsequent LC-MS analysis, as described in Materials and Methods. Briefly, 4 µL of 15 µM ARN in plasma was added to 100 µL plasma samples. Two volumes (200 µL) of ice-cold ACN were added to samples for protein precipitation and 5 µL of the clear supernatant was injected directly into the LC-MS system using electrospray ionization in positive ion mode and single ion recording. Analytes and IS peaks were eluted from the column using 8% of 6.25 mM ammonium acetate buffer pH 4.5 (final concentration 0.5 mM), acetonitrile between 40% and 60% balanced to 100% with water at a flow rate of 0.4 mL/min. The ammonium-adducts of AS, DHA, and ARN were detected at mass/charge (m/z) ratios of 402 and 302, and 300, respectively. In addition to these unique peaks of analytes, common fragments gave rise to m/z peaks at 267 and 284 (Figure 1).

**Figure 2:**
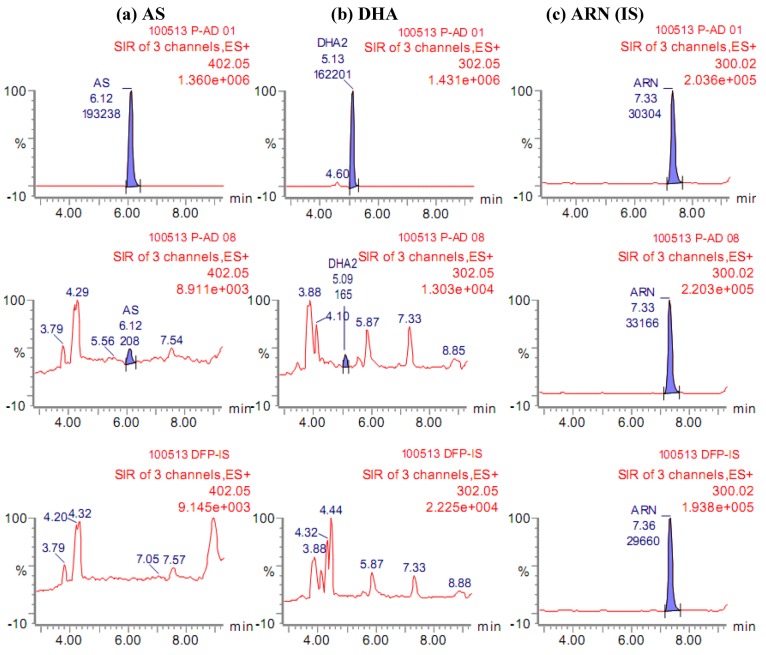
Representative chromatograms of (bottom) drug-free plasma, (middle) AS/DHA 3.20/5.33 nM at LLOQ and (top) 3,000/5,000 nM at ULOQ of (**a**) AS and (**b**) DHA spiked in plasma with (**c**) ARN 600 nM as internal standard (IS). Shaded areas indicate specific peaks used for quantification using IS method.

As can be seen in Figure 2, there were no detectable endogenous components that would interfere with AS or DHA in drug-free plasma (bottom panel). Artesunate eluted from the column at 6.12 min as a single peak (Figure 2a), whereas the α- and β-epimers of DHA eluted as two peaks at 4.6 and 5.1 min, respectively (Figure 2b), with the major β-DHA used for quantification. The early peak represents the α-DHA epimer [16]. Artemisinin eluted at 7.33 min (Figure 2c). The system suitability test of AS and DHA at 10 and 100 nM, and ARN at 200 nM (n = 10) in 50% ACN showed very good precision with coefficients of variation (CV) of ≤4.95% for AS, ≤1.73% for DHA, and ≤2.26% for ARN.

The AS and DHA concentration ranges from 3.20 to 3,000 nM and 5.33 to 5,000 nM respectively. All the concentrations quantified using peak area ratios of the analytes:IS multiplied by the concentration of ARN (600 nM) were fitted using a 2^nd^ order polynomial equation: 


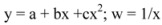
(1)


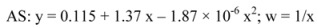
(2)


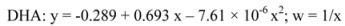
(3)

**Figure 3:**
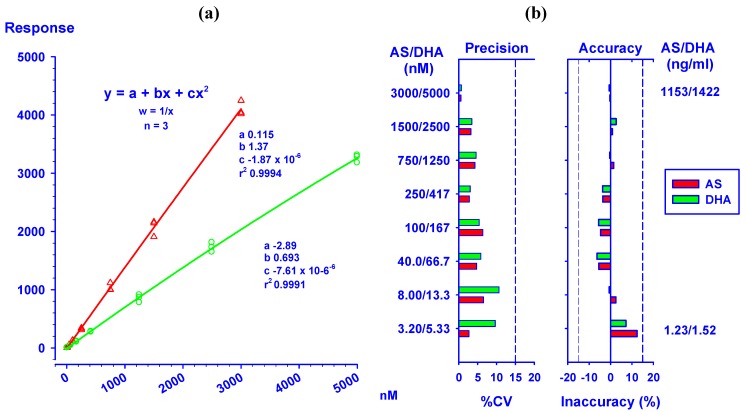
(**a**) Linearity test of the standard curves (n = 3) of AS (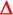)/DHA (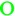) at concentrations ranging from 3.20/5.33 to 3,000/5,000 nM. (**b**) Precision (%CV) and inaccuracy (%) data from back calculated concentrations of AS/DHA. Dotted lines indicate 15% CV and ±15% inaccuracy levels. Data from triplicate analyses on the same day are shown.

Parameters in equations 2 and 3 were from the mean of three standard curves of AS and DHA analyzed on the same day. Both analytes had standard curves across the tested concentration ranges with very good correlation (r^2^ = 0.9994 for AS and r^2^ = 0.9991 for DHA). Back calculation of the concentrations of the standards indicated high accuracy and precision; falling within ±12.4 % inaccuracy (at LLOQ) and %CV ≤10.7 (Figure 3).

**Figure 4:**
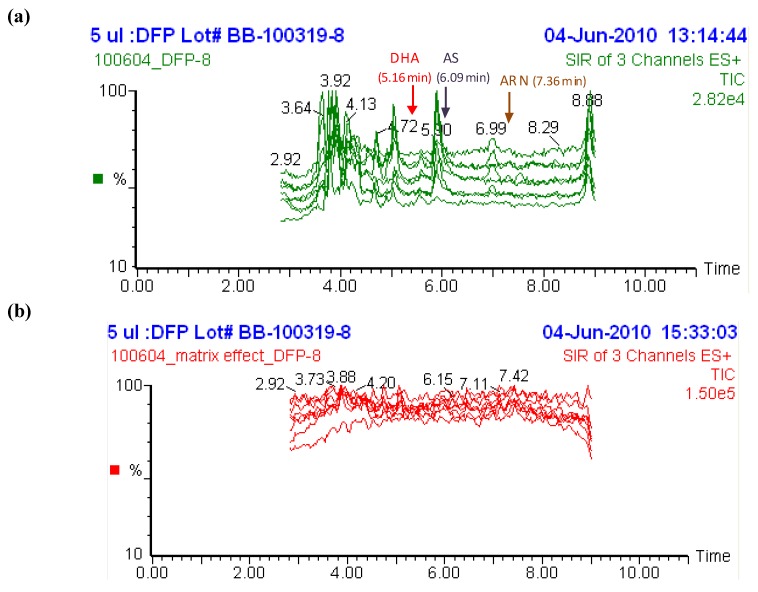
(**a**) Chromatograms of blank plasma from eight healthy volunteers. (**b**) Post-column infusion of 100 nM of AS, DHA and 200 nM of ARN at 10 µL/min during injection of blank plasma extract from the same volunteers.

The limits of detection (LOD) were determined using blank plasma samples from six donors with a signal/noise ratio of 3:1, resulting in a LOD of 1.02 nM (0.39 ng/mL) for AS and of 0.44 nM (0.13 ng/mL) for DHA. The lower and upper limits of quantification (LLOQ and ULOQ) of AS were 3.20 and 3,000 nM (1.23 and 1,153 ng/mL) and DHA were 5.33 and 5,000 nM (1.52 and 1,422 ng/mL) respectively (Table 1). No carryover into the next analytical sample was observed at 3,000/5,000 nM (as defined by ≤5% of LLOQ, data not shown). In addition, the intra-assay and inter-assay accuracy and precision (n = 6) of our assay was very good with an inaccuracy of ±7.3% and CVs of ≤8.9% at 10.0/16.0, 500/800, 2,500/4,000 and 3,000/5,000 nM for AS/DHA (Table 1). At the LLOQ for AS/DHA (3.20/5.33 nM), the intra-assay and inter-assay inaccuracy and precision were within 6.0% and ≤17.6%, respectively. The recovery of AS and DHA, from plasma samples at 50, 500 and 2,500 nM was 95–98%, and 95–115%, respectively. The recovery of ARN at 500 nM was 105%. There was no matrix effect from plasma extract during post-column infusion of AS and DHA (100 nM), and ARN (200 nM) at 10 µL/min in the region where they eluted (Figure 4). Thus, the assay for AS and its metabolite DHA is simple, accurate and precise with a low detection limit and a wide detection range.

**Table 1:** Intra-assay and inter-assay accuracy and precision tests for AS and DHA in human plasma.

	Theo. Conc .		Found (n = 6)		Inaccuracy (%)		%CV	
	AS	DHA	AS	DHA	AS	DHA	AS	DHA
Intra-assay								
1. LLOQ	3.20	5.33	3.02	5.53	-5.73	3.66	17.6	7.15
2. 3 x LLOQ	10.0	16.0	10.7	16.3	7.12	1.71	2.76	5.29
3. Medium	500	800	533	796	6.62	-0.55	4.00	5.28
4. 0.8 x ULOQ	2,500	4,000	2,481	3,810	-0.77	-4.74	1.68	3.11
5. ULOQ	3,000	5,000	2,782	4,637	-7.28	-7.27	3.78	4.10
Inter-assay								
1. LLOQ	3.20	5.33	3.28	5.65	2.36	5.93	5.44	10.2
2. 3 x LLOQ	10.0	16.0	9.93	16.7	-0.69	4.25	8.88	5.19
3. Medium	500	800	496	785	-0.76	-1.82	7.98	5.87
4. 0.8 x ULOQ	2,500	4,000	2,445	3,872	-2.21	-3.20	5.48	4.89
5. ULOQ	3,000	5,000	2,912	4,966	-2.93	-0.68	5.03	5.24

Values are average of six replicates for intra-assay test and triplicates per day for six different days of inter-assay test.

The sample dilution was tested using series of 10-fold dilutions. The dilution had no effect on the accuracy and precision measurement of both AS and DHA at 20 and 200 µM. The %CV and inaccuracy (%) of AS were <7.5 and within ±5.5, respectively. The %CV and inaccuracy (%) of DHA were <5.0 and within ±13.6, respectively.

### 2.2. Selectivity of the assay and interference from other antimalarial drugs

**Figure 5:**
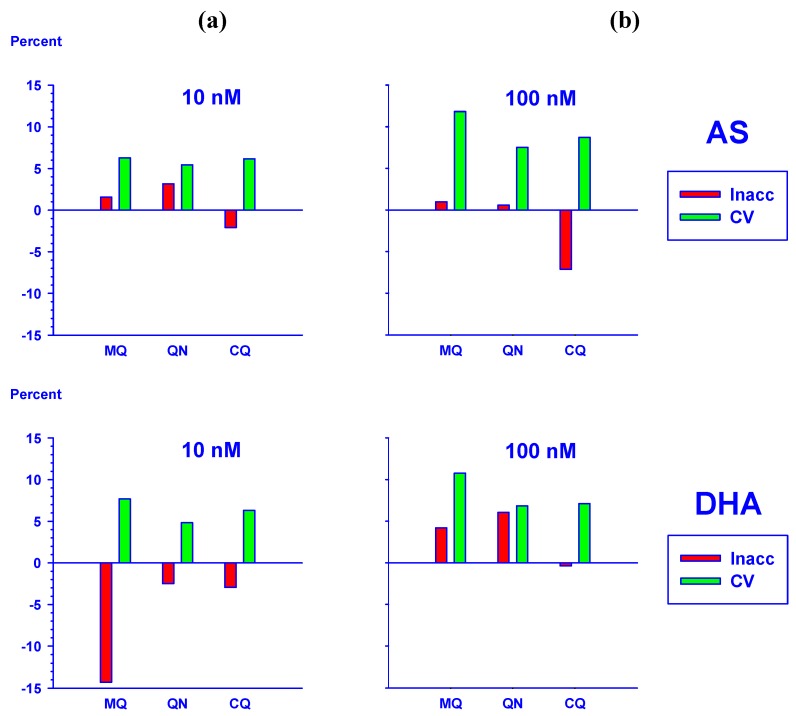
No interference by common antimalarial drugs was observed in the quantification of AS and DHA. Mefloquine (MQ), quinine (QN) and chloroquine (CQ) at 1 µg/mL did not interfere with the quantification of AS and DHA at (**a**) 10 nM and (**b**) 100 nM in plasma. The precision (%CV) and inaccuracy (%) data of 6 independent experiments are shown.

We tested if the assay was selective for AS and DHA. Endogenous substances interfering with AS and DHA were not detected in drug-free plasma (Figure 4a) from eight healthy volunteers (as defined by ≤5% of LLOQ, data not shown). Moreover, interference of other antimalarials commonly used, namely mefloquine (in combination with artesunate), quinine and chloroquine, were evaluated by addition of these drugs at 1 µg/mL to AS and DHA standards in plasma. No interference by these antimalarial drugs was observed for either AS or DHA at 10 and 100 nM as indicated by <15% CV and values within ±15% inaccuracy (Figure 5).

### 2.3. Post-preparative stability test of DHA and AS

#### 2.3.1. Dihydroartemisinin epimerization

**Figure 6:**
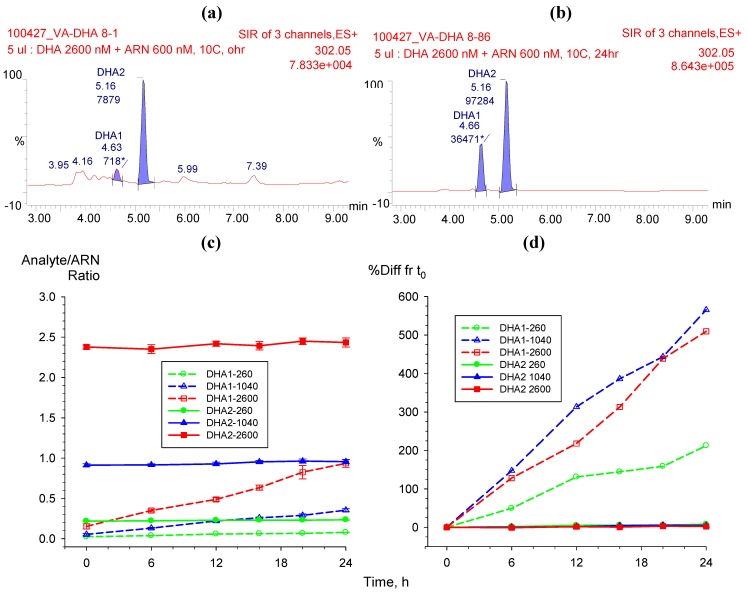
Post-preparative stability test of DHA in autosampler (10 °C) over 24 h period showing chromatograms (top panel) of α-DHA (t_R_ 4.6 min) and β-DHA (t_R_ 5.16 min) at (**a**) time 0 and (**b**) 24 h. (**c**) The mean (±95%CI) responses of five replicate analyses and (**d**) the % difference from t_0_ of both α- and β-epimers.

Dihydroartemisinin undergoes epimerization as α and β epimers in solution. In 50% ACN solution β-DHA is the major epimer, with minute amount of α-epimer. The retention time (t_R_) for α-DHA was 4.6 min and for β-DHA was 5.16 min (Figure 6a,b). The presence of α-DHA and β-DHA epimers were evaluated after extracting from plasma as described. The supernatant was used for post-preparative stability test of DHA in an autosampler (10 °C) over a 24 h period. In plasma extracts, β-DHA was still the major epimer. The responses of β-DHA at 260, 1,040, and 2,600 nM (n = 5) showed a slight increase; up to 7.8% compared to initial concentration measured at time zero (t_0_) which is within acceptable range for stability test (Figure 6c,d). In contrast, α-DHA , the minor epimer in plasma extracts, showed a steady increase up to 600% difference from t_0_ (Figure 6d). Therefore, the major peak β-DHA was used for quantification of DHA. 

The present study is the first to assess the stabilization of the α- and β-epimers of DHA at 6 then 4 h intervals over 24 h following plasma extraction. β-DHA at 260, 1,040, and 2,600 nM was quite stable with ≤7.8% differences in concentration compared with t_0_ values. In contrast, α-DHA concentrations increased steadily over 24 h with up to a 600% difference in concentration compared with the t_0_ value. Thus, because of β-DHA’s larger peak area and greater stability, it was used for the quantification of DHA. Table 2 summarizes several recent LC-MS or LC-MS/MS methods for the quantification of AS and DHA in human plasma, including our method. Others have reported α-DHA as the major epimer, with the α:β ratio stablising after equilibration at 4 °C for 15 to 18 h [9,13], with ratios varying between 3.8 and 6 [10,11,12]. Hanpithakpong *et al*. [13], however, were only able to detect α-DHA using the same ionization source. The disparity between our findings with β-DHA being the major epimer and others [16,17,18,19] where α-DHA is the predominate isomer may be due to differences in column temperature, solvent composition and chromatographic conditions [16,17]

#### 2.3.2. Hydrolysis of AS to DHA

**Figure 7:**
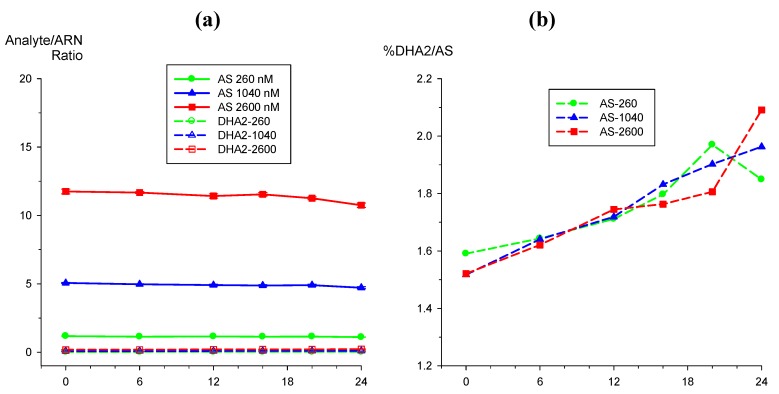
Post-preparative stability test of AS at 260, 1,040, and 2,600 nM in autosampler (10 °C) measured at 6 then 4 h intervals over a 24 h period showing (**a**) AS/IS and β-DHA/IS ratios and (**b**) β-DHA formation due to hydrolysis of AS.

Post-preparative stability test of AS (alone, n = 5) at 260, 1,040, and 2,600 nM in an autosampler (10 °C) over a 24 h period showed that AS reponses were quite stable with ratios changing by <−8.6% from t_0_ values (Figure 7a). The response of β-DHA, formed by auto-hydrolysis of AS, was less than 2.1% (Figure 7b). Therefore, combination of AS and DHA were used to construct standard curves and QC samples for quantification of analytical samples where both AS and DHA are present.

**Table 2:** Summary of recent analyses of AS and DHA in human plasma using LC-MS or LC-MS/MS compared to current method.

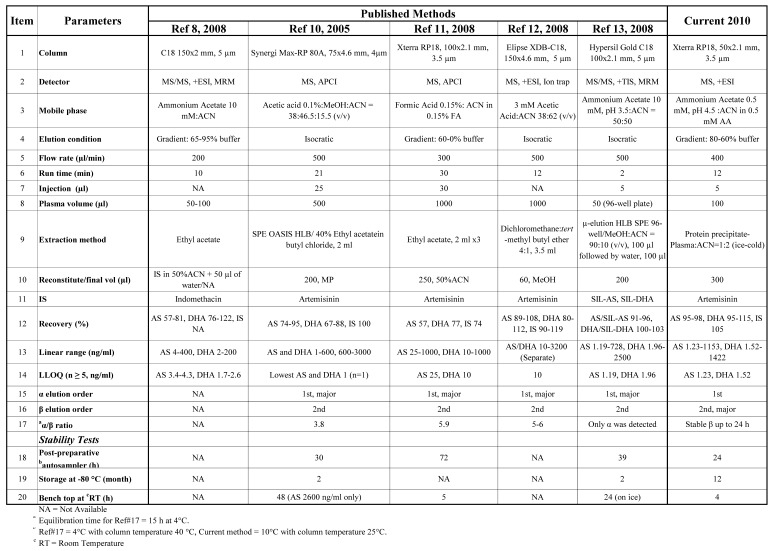

### 2.4. Stability test of AS and DHA in plasma samples

There was no significant decrease in the amount of AS and DHA detectable over time at 50 nM and 2,500 nM. At room-temperature (19–25 °C), AS and DHA in plasma remained stable for up to 6 h (Table 3). In addition, after protein precipitation using ACN, both compounds were stable up to 72 h when refrigerated at 2–8 °C. Moreover, the concentrations of AS and DHA were not influenced by three freeze/thaw (-80 °C/RT) cycles (Table 3).

Table 3Stability tests of AS and DHA in plasma (**a**) at room-temperature and after three cycles of freezing/thawing, and (**b**) at long-term storage temperature (-80 °C).

**Table:** (**a**)

		50 nM		2500 nM	
		AS	DHA	AS	DHA
Room Temperature (Fresh)	0 h	52.0	54.5	2490	2547
		*(0.00, 1.15)*	*(0.00, 2.11)*	*(0.00, 13.8)*	*(0.00, 9.42)*
	2 h	46.2	50.2	2256	2292
		*(-11.2, 0.88)*	*(-1.67, 3.20)*	*(-9.40, 10.6)*	*(-10.0, 7.84)*
	4 h	44.1	46.9	2250	2243
		*(-15.3, 1.84)*	*(-8.14, 2.69)*	*(-9.64, 4.23)*	*(-11.9, 2.27)*
	6 h	44.5	46.4	2267	2279
		*(-14.4, 3.20)*	*(-9.06, 3.46)*	*(-8.96, 4.45)*	*(-10.5, 0.49)*
Freeze/Thaw Cycles	0 cycle	47.0	50.0	2606	2467
		*(0.00, 9.76)*	*(0.00, 9.96)*	*(0.00, 1.86)*	*(0.00, 1.33)*
	3 cycles	50.3	53.2	2392	2384
		*(7.01, 3.75)*	*(6.39, 0.99)*	*(-8.23, 0.79)*	*(-3.35, 1.34)*

Values represent mean (%Difference from t_0_, %CV) of three independent measurements

**Table:** (**b**)

Storage Duration	50 nM		2500 nM	
(month)	AS	DHA	AS	DHA
0 (Fresh)	51.4	53.2	2567	2561
	*(0.00, 2.22)*	*(0.00, 3.74)*	*(0.00, 9.15)*	*(0.00, 6.07)*
1	52.5	50.7	2539	2370
	*(2.22, 11.2)*	*(-4.61, 10.3)*	*(-1.10, 8.52)*	*(-7.46, 7.85)*
2	48.5	47.7	2454	2227
	*(-5.66, 5.98)*	*(-10.2, 3.17)*	*(-4.40, 7.95)*	*(-13.1, 10.7)*
6	42.7	47.7	2501	2393
	*(-16.8, 2.17)*	*(-10.3, 9.22)* *	*(-2.56, 9.93)*	*(-6.58, 2.33)*
12	55.0	47.0	2547	2174
	*(7.04, 10.2)*	*(-11.5, 15.8)*	*(-0.80, 15.2)*	*(-15.1, 13.0)*

Values represent mean (%Difference from t_0_, %CV) of six independent measurements (* n = 5)

**Figure 8:**
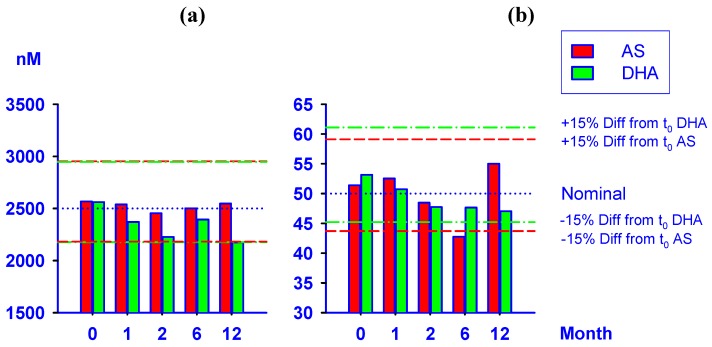
Stability of AS and DHA in plasma at (a) 2500 nM and (b) 50 nM when stored at -80 °C over 12 months. Mean values based on six replicate analyses with dashed lines showing ±15% differences compared to initial concentration measured at time zero (t_0_).

Previously reported data on the stability of AS in patient samples using LC-MS [10] and LC-MS/MS [13] methods have only extended out to two months of storage. This is often inconvenient or logistically difficult in clinical malaria field studies. We report plasma samples containing AS or DHA analyzed after storage at −80 °C for 1, 2, 6, and 12 months. Figure 8 shows mean of six replicate analyses indicating that both analytes were highly stable under these conditions for the duration of the study. Less than 15% inaccuracy was observed in measurement at the respective concentration-time points compared to the concentration at t_0_ except for AS 50 nM (−16.8%) after 6 months and DHA 2,500 nM (−15.1%) after one year of storage. Similarly, the %CV values were <15% except for AS 2,500 nM (15.2) and DHA 50 nM (15.8) after one year of storage.

To date, only one study using HPLC-ECD reported storage stability test up to 12 months; but, the accuracy tests were within ±15% up to 6 months [9] only. In practice, the time interval between collected clinical samples and analyses is often greater than 2 months and often carries out to one year.

### 2.5. Application to clinical sample analysis

#### 2.5.1. Effect of haemoglobin on the quantification of AS and DHA

**Figure 9:**
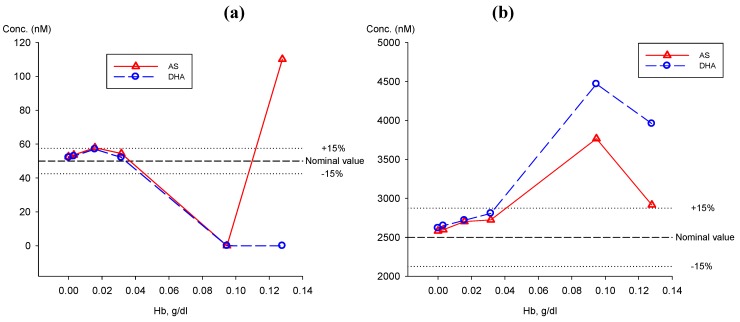
Effect of haemoglobin (Hb) from haemolyzed blood in plasma on the measurement of AS (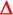) and DHA (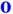) at (**a**) 50 nM and (**b**) 2,500 nM.

Haemoglobin (Hb) levels in plasma arbitrarily classified as 0, +1, +2, +3, +4, and +5 were 0.00, 0.0032, 0.0158, 0.0316, 0.0947, and 0.1278 g/dL respectively. The average (n = 3) of AS and DHA concentrations found at 50 and 2,500 nM were plotted against its Hb levels and are shown in Figure 9. At both AS and DHA concentrations, the measurement of AS and DHA were not affected by the presence of Hb up to 0.032 g/dL (+3) compared to control (Hb = 0). The % difference was <10% and 7.2% at 50 and 2,500 nM of AS/DHA, respectively. The measurement of AS and DHA was greatly unpredictable by the presence of plasma Hb > 0.09 g/dL.

#### 2.5.2. Analysis of AS and DHA in plasma samples from a clinical trial

**Figure 10:**
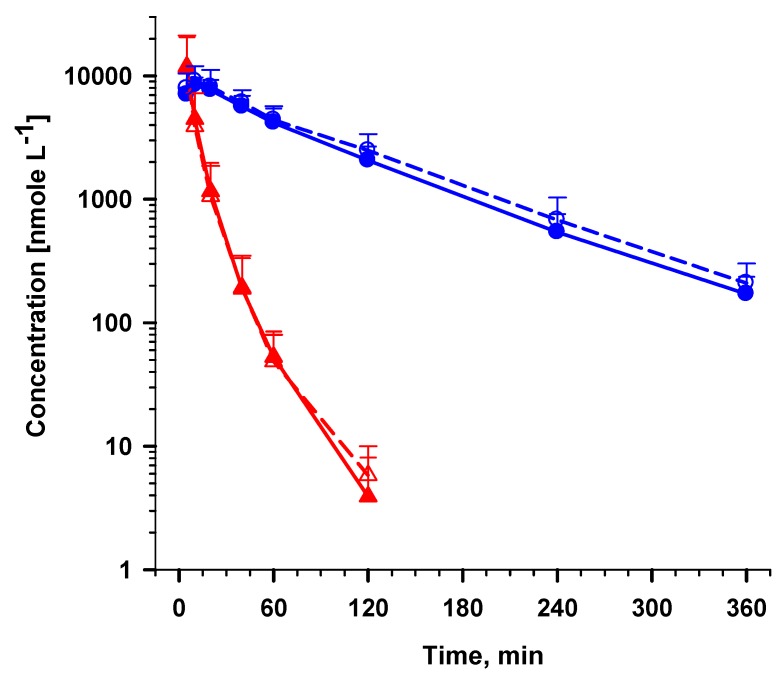
Application to clinical plasma samples in plasma from three patients following IV AS treatment (2.4 mg/kg) using the validated assay and cross-validation of AS (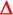) and DHA (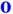) by external standard (solid lines) method compared to internal standard method using ARN as IS (broken lines). Values are mean + SD.

This method was deemed suitable for the analysis of AS following an IV AS administration where 20–30 µM of AS was reported five min post-dose of AS at 120 mg in Vietnamese patients with uncomplicated and severe malaria [18,19]. The concentration of AS was as high as 200 µM at five min post-dose following an IV AS dosage of 8 mg/kg in healthy volunteers [20]. Our simplified assay was used to quantify pre- and post-dose samples from African adult severe malaria patients receiving treatment with IV AS (US Army GMP formulation; IND 64,769) at 2.4 mg/kg. The human samples were collected under an IRB approved protocol where plasma was immediately separated and stored frozen at -80 °C until analysis. Figure 10 shows average + SD for three patients.

Almost none of the 300 plasma samples collected in this clinical study were contaminated with haemolysis products. The few samples showing haemolysis products were at levels (equivalent to Hb +1 to +2) that did not alter the drug concentration-time profiles, except for one (equivalent to Hb +5) sample. In this sample both plasma AS and DHA concentrations were significantly lower than expected. The use of stable isotopic labeled (SIL) AS and DHA as IS [21] may correct for highly haemolyzed samples. However, the SIL compounds are not readily available and are rather expensive. It should be noted that for laboratories registered and accepted in the World Wide Antimalarial Resistance Network (WWARN), SIL compounds as IS (including reference standards) will be issued at no cost for research purposes. The samples from this clinical study were used for cross-validation between external and internal standard methods. Unfortunately, there was not enough plasma sample with Hb +5 for cross-validation. Figure 10 clearly shows that both methods of quantification were comparable when the plasma was not contaminated with Hb > 0.09 g/dL.

## 3. Conclusions

We report a novel assay for the accurate and selective quantification of AS and its metabolite DHA in plasma samples based on one-step protein precipitation followed by LC-MS assay. Our method is simple, reproducible, sensitive, and satisfies the practical requirements of conducting antimalarial clinical trials in austere settings. The method has been validated to quantify both compounds over a concentration range (LLOQ–ULOQ) of 3.20–3,000 nM (1.23–1,153 ng/mL) for AS, with a LOD of 1.02 nM (0.39 ng/mL) and 5.33–5,000 nM (1.52–1,422 ng/mL) for DHA, with a LOD of 0.44 nM (0.13 ng/mL). The method has been validated to measure both compounds at the concentrations outside the calibration range up to 200 µM. The approach is suitable for clinical samples from patients treated with IV AS formulation. We have extended the stability data by showing that both AS and DHA in plasma remained stable for up to one year at -80 °C within ±15% of the initial concentration measured at time zero. In addition, the method described here detected β-DHA as a major epimer and showed that it was stable up to 24 h in an autosampler (10 °C). There was no requirement for extra time of more than half a day to allow equilibration of the α/β DHA epimers, as reported by others. It was observed that plasma haemoglobin levels greater than 0.09 g/dL compromised the quantification of AS and DHA. More investigation is needed to address this issue and the use of stable isotopic labeled internal standard when available may be warranted. 

## 4. Experimental

### 4.1. Reagents

Artesunate or artesunic acid (AS, WR256283, Bottle# BQ38641, MW 384.425), dihydroartemisinin (DHA, WR253997AN, Bottle# ZW60909, MW 284.355) and artemisinin (ARN, WR49309, Bottle# BN97453, MW 282.332) as well as mefloquine (MQ), quinine (QN) and chloroquine (CQ) were obtained from the Experimental Therapeutics Chemical Inventory System of the Walter Reed Army Institute of Research (Silver Spring, MD, USA). Artesunate and DHA had purities of 99.5% and 98%, respectively. Acetonitrile (Optima grade) was purchased from Fisher (Waltham, MA, USA), glacial acetic acid (HPLC grade) from J.T. Baker (Phillipsburg, NJ, USA) and ammonium acetate (99.999%) was obtained from Aldrich (St. Louis, MO, USA). Ultra-pure analytical grade Type 1 water was obtained from Simplicity 185 Water Purification System (Millipore Corporation, Billerica, MA, USA) freshly before use for the preparation of standards and aqueous solutions. Outdated human plasma (Lot# BB-100319) was obtained from a local blood bank using whole blood with citric phosphate dextrose, anticoagulant (Red-cross, Bangkok, Thailand). De-identified patient samples for method validation were kindly obtained from Colonel Peter J. Weina from the Walter Reed Army Institute of Research (Silver Spring, MD, USA).

### 4.2. Instrumentation/Equipments

The chromatographic system consisted of a reversed phase column (XTerra MS C18, 3.5 µm, 2.1 × 50 mm) and a pre-column of the same material (2.1 × 10 mm) mounted on an Alliance 2695 Liquid Chromatography System (autosampler set at 10 °C and column compartment set at 25 °C) equipped with a single quadrupole Micromass ZQ (MM1) mass spectrometry detector and MassLynx/QuanLynx^®^ v.4.0 software was used for data acquisition and quantification (Waters Corporation, Milford, MA, USA). A nitrogen generator (NM30LA, Peak Scientific, Billerica, MA, USA) was used with the MS system for cone gas flow. A micro-analytical balance (AX-26 Comparator, MettlerToledo, Columbus, OH, USA), and a high-speed centrifuge (Eppendorf 5415D or 5417R, Hamburg, Germany) were used for the preparation of standards and samples. Haemoglobin was measured in whole blood by Sysmex XT2000i (Chuo-ku, Kobe, Japan). A −80 °C freezer (Revco ULT 2586-9-V38, Waltham, MA, USA) with circular temperature recording pen was used for long-term storage of analytical samples. The freezer was physically checked once daily (except weekends) and the recording pad changed weekly.

### 4.3. Standards

#### 4.3.1. Standard curves

Stock solution of AS, DHA and ARN (used as internal standard) of 5 mM were prepared in 100% ACN, serially diluted in 50% ACN and finally in human plasma or 50% ACN (for neat drug analysis). All the standards were accurately weighed on a micro-analytical balance. Combined AS and DHA working solutions were prepared in 50% ACN so that when spiked in human plasma (1/25 dilution) the resulting final AS/DHA concentrations were 3.20/5.33, 8.00/13.3, 40.0/66.7, 100/167, 250/417, 750/1,250, 1,500/2,500, 3,000/5,000 nM. Stock solutions of other antimalarials were prepared at 10 µg/mL and diluted to 1 µg/mL in 50% ACN solution. All solutions were prepared in polypropylene tubes and micro-centrifuge tubes. The stock solutions were stored in -80 °C freezer.

#### 4.3.2. Quality controls

The working solutions for quality control were prepared from the same stock solution as the standard curve. The working solutions were freshly diluted 25-fold each, using the same sample matrix as that of the standard curve. The final concentrations of AS/DHA in the solutions for analysis were 10.0/16.0, 500/800, and 2,500/4,000 nM. Each quality control solution was prepared in triplicate and analyzed. 

#### 4.3.3. Internal standard

The working solution of ARN at 200 µM in 50% ACN was diluted to 15 µM using human plasma. The resulting solution (4 µL) was added to all the samples and vortex before analysis. The total %ACN in plasma sample was less than 2%.

### 4.4. Sample preparation

All standards, QC and analytical samples in either plasma or 50% ACN solution were handled in the same manner, using Eppendorf micro-centrifuge tubes. Artemisinin (15 µM, 4 µL) was added as IS to all samples (100 µL) after which the drugs were extracted from plasma by protein precipitation using 2 volumes of ice-cold ACN (e.g. 100 µL of sample and 200 µL of ACN), vortex for 1 min, and centrifugation at 10,000 RPM for 10 min. A 100 µL aliquot of the clear supernatants were transferred to HPLC glass vials from which the autosampler made 5 µL injections into the LC-MS system (The remaining solution was stored in the refrigerator for later repeat analysis within 24 h when necessary). For the 50% ACN matrix, the sample solution was diluted 3-fold with two volumes of ACN before injecting 5 µL into the LC-MS system.

### 4.5. LC-MS conditions

The mobile phase contained 6.25 mM ammonium acetate, pH 4.5 and the buffer-acetonitrile-water was delivered via the LC’s quaternary gradient system. The buffer contribution was fixed at 8% with initial mobile phase composition of 20% ACN and the remaining volume made up with water. The initial composition was held for 1 min to remove polar solvent peaks, changed to 40% ACN in 0.5 min, held for 4.5 min to elute both analytes, changed to 60% ACN in 1 min, held for another 1 min to elute the ARN, changed back to 20% ACN in 1 min and held for the last 2 min to equilibrate the column for following run. The flow rate was at 0.4 mL/min throughout the analysis time of 12 min (including injection delay). 

The mass spectrometer was set in the positive electrospray ionization mode and single ion recording. The parameters were optimized for each analyte and IS by infusion of 500 ng/mL in initial mobile phase composition at 10 µL/min. The voltage for capillary, cone, extractor and RF lens were set at 0.60 (kV), 12, 1.00, and 0.4 V respectively. The source and cone temperature were set at 120 and 20 °C. The nitrogen generator was set at 100 psi to generate a cone and desolvation gas flow set at 30 and 300 L/h respectively. For the analyzer, both LM1 and HM1 resolution were set at 13 with the ion energy set at 0.6 and the multiplier set at 650 V.

### 4.6. Quantification method

For quantification, the peak areas of AS, DHA and ARN were integrated using the MassLynx/QuanLynx chromatography software. The response was calculated from the ratio of the peak areas of the analyte:IS multiplied by the concentration of IS and plotted against concentration in plasma. The responses-concentrations of the standard curve were fitted to the polynomial equation including origin using 2^nd^ order curve type and weighting (Equation 1). Least square regression analysis was performed and the resulting parameters were used to back calculate the concentration of the standard and calculate the concentration of the samples.

### 4.7. Validation

Full validation [10,11] was performed without IS. After modifying the method by using ARN as IS, a partial validation was performed for system suitability, recovery, matrix effect, linearity, LLOQ, ULOQ, intra-assay and inter-assay variability for accuracy and precision, post-preparative stability of AS and DHA, effect of haemoglobin (Hb) on the measurement of AS and DHA, and cross-validation using clinical plasma samples following IV AS (2.4 mg/kg).

#### 4.7.1. System suitability

The system suitability test was done using solutions of 10 and 100 nM to each analyte and 200 nM IS prepared in 50% ACN solution. The solutions were injected in consecutive sequences of ten replicates. The peak area responses were measured and the injection precision (%CV) determined.

#### 4.7.2. Selectivity

Human plasma from six donors were extracted and analyzed for endogenous peak that might interfere with the analytes peak. The detection limit (LOD) was determined at the same time by determining the noise near the region where the analytes eluted using the average noise multiplied by 3. The resulting response was then extrapolated from the standard curve to calculate the concentration at LOD. Interference from other xenobiotics (such as antimalarials: mefloquine, quinine and chloroquine) often co-administered or used in combination with AS were evaluated at 1 µg/mL in 50% ACN. When there was no interference in the neat drug solution, the drugs were spiked in plasma with the analytes at 10 and 100 nM and analyzed in six replicates. 

#### 4.7.3. Linearity test

Standard curves were prepared using triplicates of each AS/DHA concentration in plasma covering the range of at 3.20/5.33 to 3,000/5,000 nM. The analysis was done using ARN as IS at 600 nM and performed one curve at a time. The response of each analyte was obtained from the peak area ratio of the analyte:IS and multiplied by the concentration of IS. The standard curve was constructed by plotting the response against its concentration in plasma, linearly fitted, and weighed by 1/concentration. The linear equation parameters of the standard curve were reported as an average of 3 individual curves. 

#### 4.7.4. Accuracy and precision

The intra-assay and inter-assay accuracy and precision of the method was evaluated at 5 different AS/DHA concentrations in plasma: LLOQ, 3xLLOQ, medium, 80% of ULOQ and ULOQ. Six replicates for each concentration were measured for the intra-assay test. Triplicate analyses per concentration per day over different days were analyzed for the inter-assay test. The concentrations of AS/DHA were 3.20/5.33, 8.00/13.3, 10.0/16.0, 500/800, 2,500/4,000, 3,000/5,000 nM. The accuracy was determined by the deviation of the mean value of the found concentration from the theoretical value. The coefficients of variation for each tested concentration was calculated to determine the precision. Following the analysis of the highest concentration (ULOQ–3,000/5,000 nM), an injection of 50% ACN was run to evaluate carry-over effect. For concentration over the ULOQ, the sample dilution with the same matrix as the sample was evaluated at 20 and 200 nM in plasma by performing a series of 10-fold dilution until the concentration was in the working range – between LLOQ and ULOQ. The analytes were extracted from plasma samples and analyzed in six replicates.

#### 4.7.5. Recovery and matrix effect

The recovery was determined at three concentrations: 50, 500, and 2,500 nM of AS and DHA spiked in 50% ACN, and compared to plasma samples. The sample process and analyses of analytes were performed in the same manner for both matrixes. The response of the analytes in 50% ACN was considered as 100%. The matrix effect from plasma of eight donors was evaluated by injecting the extracted plasma samples during post-column infusion of the analytes (100 nM) and IS (200 nM) in 50% ACN at 10 µL/min.

#### 4.7.6. Stability

All stability determinations were performed by using a set of samples prepared from a freshly made stock solution of both analytes in analytes-free, interference-free plasma. The stock solution of both analytes was diluted in 50%ACN and spiked in the blank plasma, in triplicates, at 50 and 2,500 nM. The analytes in spiked plasma samples were then extracted and analyzed in triplicates per concentration except where specified. The test and freshly prepared solution were then compared. 

Stability of stock solution in 50% ACN was determined in three sets of aliquots stored - at room temperature (25 °C) for 6 h, refrigerated temperature (8 °C) for 1 and 2 weeks, and frozen temperature (−80 °C) for 1 and 2 months. Post-preparative stability was determined by extracting five aliquots of the spiked plasma samples at each concentration, the supernatant was then stored in the autosampler (10 °C) for 0, 6, 12, 16, 20, and 24 h. The post-preparative stability tests were performed for each analyte alone to evaluate hydrolysis of AS to DHA and its epimerization to α-DHA and β-DHA. The test was also evaluated in the combination of AS/DHA.

In plasma, freeze/thaw stability of AS/DHA was evaluated by using three aliquots of the spiked plasma samples at each concentration, stored at −80 °C for 24 h and completely thawed unassisted at room temperature. The analytes in spiked plasma samples were extracted and analyzed after the samples underwent up to three cycles. The short-term room temperature (bench top) stability was tested at 0, 2, 4, and 6 h. Spiked plasma samples were extracted, analyzed and quantified against freshly prepared standard curves in plasma. For long-term storage stability test, the spiked plasma samples in six replicates were stored at -80 °C for 0, 1, 2, 6, and 12 months and followed the same procedure as the short-term stability test. 

### 4.8. Application to clinical plasma samples

Blood samples from severe malaria patients are sometimes haemolyzed. The haemolytic products related to sample collection and/or malaria infection may degrade artemisinin derivatives [20]. Since the major haemolytic product is Hb, its effect on the measurement of AS and DHA was evaluated. 

The Hb level in whole blood was measured before separation of plasma and after replacing the plasma with the exact same volume of water and vortexing for 5 min to lyse the red blood cells (RBC). The Hb level in the lysed RBC (100% RBC) was the same as that of whole blood. The 100% RBC was serially diluted with water to 40, 30, 10, 5, and 1% (v/v) and 14 µL of each %RBC suspension was spiked into 0.7 mL plasma to generate five different levels of Hb in plasma samples as to cover the range of colors seen in clinical samples (+1 to +5). The Hb levels in plasma were calculated from the original level in 100%RBC. At each Hb level in plasma (including control without Hb) AS and DHA were spiked in the plasma at 50 and 2500 nM in triplicates per concentration. The analytes were then extracted, and analyzed by LC-MS.
